# The Nursing Mirror

**Published:** 1888-04-07

**Authors:** 


### The Nursing Mirror.

Communications for this column should be addressed The Mirror, care of the Editor, 38, Old Jewry, London, E.C.

### THE BRITISH NURSES ASSOCIATION.

I fully agree with "F. H. M." in many of her remarks. The chief object aimed at in the proposed registration of nurses is, I believe, to protect duly-qualified nurses, and to raise the position of those who are thoroughly trained. As "F. H. M." says, how can this be done if every "Sairey Gamp" can be enrolled on the register, provided she pays her subscription before January, 1889? I think that every nurse should, before she can be registered, either hold a certificate from some hospital or training institution, where probationers are trained, or else should be required to pass an examination in both practical and theoretical nursing, including a little elementary physiology and hygiene. I think it should also be essential that she should be able to pass in reading, writing, and spelling, at any rate Standard VI. Surely the "theory of nursing" will some day be one of the subjects for which certificates will be given in the Science and Art Department at South Kensington! Why not, as well as the "theory of music," "hygiene," and "physiology"? Then it would be an easy matter for candidates to come up to an examiner for *viva voce* and practical demonstration, just as they do now in practical chemistry, &c.

A Workhouse Infirmary Superintendent of Nurses.

London.

### A PLEA FOR THE NURSES.

You would be doing a real service to the nurses and probationers of ——— Hospital, if you would call the attention of the governors to the following deficiences in organization, which they would, no doubt, remedy at once, if they were aware of them. (1) In most large hospitals the heavy medicine trays and stimulant baskets are sent upstairs by lifts or are carried by porters at ——— Hospital the probationers themselves have to toil up the long flight of stone steps, loaded almost beyond their strength; (2) All the soiled linen and dressings are carried by the nurses from the top of the building to the basement, instead of being sent down a long shoot, as in other hospitals; (3) The addition of noiseless castors to the heavy screens put round the beds, would save the nurses much unnecessary labour; (4) The lavatories and sculleries are not yet lighted with the long talked of gas; (5) If from any cause a nurse is prevented from getting a meal at the proper time she must go without it. Surely this is unreasonable, and cannot be known to the governors of the hospital.

S. B.

[We have communicated with the authorities of the Hospital mentioned in S. B.'s letter, and are informed that the statements contained in the letter are not in accordance with fact.—Ed. *T. H.*]

### EXPLANATIONS AND SUGGESTIONS.

When I expressed an opinion last week in favour of "The British Nurses Association" I did not understand it to be in opposition to the Hospitals Association. It would be silly and foolish to join any society for that purpose. As a nurse, distinguished from a matron or sister, with about the average amount of knowledge possessed by most others in the same position in a general hospital, I may venture to state that very little is actually understood or known on the subject of associations, and it is quite likely many would hardly be able to from a wise or sensible opinion about which would be the best. The reasons in favour of any association with us are simple enough. Some of which are: The prevention of unqualified, incompetent persons being able to pass as fully-trained nurses without having taken the trouble and care necessary to gain the experience required to prevent them being a disgrace to the profession. Also grievances might receive due consideration and be fairly dealt with. Means for mutual improvement might be found. By associating, strength and boldness might be gained, which at present seem wanting. Nurses of my own class are very timid, and dread, as a rule, notoriety in anything, enduring anything rather than create a stir in their own cause, however needed. As we are a large body really, and the real workers immediately about the sick, we deserve consideration. I think it would be well if more pains were taken by matrons and others who employ nurses to explain a great many things of importance to them to know, and draw to from them their opinions so as to prevent them mistaking one society for another, or joining any rashly.

A Constant Reader.

### NURSING IN INDIA.

Would the Editor or any reader be so kind as to give a trained, certificated nurse information as to where it would be best to apply to obtain nursing employment in India.

E. R. S.

### INVALID HOME AND TRAINED NURSES' INSTITUTION.

Could you, or any of your readers, inform me, through the medium of your columns, where a first-class invalids' home and trained nurses' institution would answer? Is there any favourite resort for invalids where there is not one already established?

Lady Superintendent.

### NURSING HOME AT CAIRO.

Sir Sydney Waterlow, who has been travelling in Egypt, has carried with him all his practical sympathy with hospitals, and the sick who resort to them. He has instituted a scheme for the establishment of a nursing home in connection with the Kasr-el-Aini Egyptian Hospital in Cairo. The scheme, which will include the establishment of two highly-educated and trained English nurses as superintendents and instructresses of the native Egyptian nurses, is receiving the warm support of the Khedive, and is specially appreciated by the European residents in the city. It is hoped that a beginning of work may be made at the end of the current month or the beginning of May. At the commencement the whole management of the home is proposed to be vested in the senior medical officer of the Kaisr-el-Aini, Dr. Milton, with a proviso that should the number of nurses so increase as to render it advisable, a board of trustees may be elected from the subscribers to the fund. The Khedive imposes two regulations as the conditions of his support and encouragement—first, that the English nurses shall on no account attempt to make proselytes among the native patients; and second, that no female nurse shall attend a male patient. Both these conditions have been accepted.

